# Homophily in Personality Enhances Group Success Among Real-Life Friends

**DOI:** 10.3389/fpsyg.2020.00710

**Published:** 2020-05-04

**Authors:** Michael Laakasuo, Anna Rotkirch, Max van Duijn, Venla Berg, Markus Jokela, Tamas David-Barrett, Anneli Miettinen, Eiluned Pearce, Robin Dunbar

**Affiliations:** ^1^Department of Digital Humanities, Cognitive Science Unit, University of Helsinki, Helsinki, Finland; ^2^Population Research Institute, Väestöliitto, Helsinki, Finland; ^3^Leiden Institute of Advanced Computer Science (LIACS), Leiden University, Leiden, Netherlands; ^4^Institute of Criminology and Legal Policy, University of Helsinki, Helsinki, Finland; ^5^Universidad del Desarrollo, Santiago, Chile; ^6^Trinity College, University of Oxford, Oxford, United Kingdom; ^7^Väestöliitto Population Research Institute, Helsinki, Finland; ^8^National Folk Pention Institution, Helsinki, Finland; ^9^Division of Psychiatry, University College London, London, United Kingdom; ^10^Department of Experimental Psychology, University of Oxford, Oxford, United Kingdom

**Keywords:** friendship, personality, Big Five, groups, group performance, inclusion-of-other-in-self

## Abstract

Personality affects dyadic relations and teamwork, yet its role among groups of friends has been little explored. We examine for the first time whether similarity in personality enhances the effectiveness of real-life friendship groups. Using data from a longitudinal study of a European fraternity (10 male and 15 female groups), we investigate how individual Big Five personality traits were associated with group formation and whether personality homophily related to how successful the groups were over 1 year (*N* = 147–196). Group success was measured as group performance/identification (adoption of group markers) and as group bonding (using the inclusion-of-other-in-self scale). Results show that individuals’ similarity in neuroticism and conscientiousness predicted group formation. Furthermore, personality similarity was associated with group success, even after controlling for individual’s own personality. Especially higher group-level similarity in conscientiousness was associated with group performance, and with bonding in male groups.

## Introduction

Social relations with peers are essential for survival, health and well-being (Caccioppo, 2008; David-Barrett and Dunbar, 2017) and the challenge of choosing friends and maintaining relations with them is one of our main social tasks today (Pearce, 2014). Friendship formation is known to often follow principles of homophily, or attraction to similarities, as shown in relation to social, ethnic and gender characteristics (e.g., McPherson et al., 2001; Launay and Dunbar, 2015; David-Barrett, 2020), life stage (e.g., David-Barrett et al., 2016), beliefs and interests (Curry and Dunbar, 2013a, b), genotypes (Fowler et al., 2011), and personality (e.g., Selfhout et al., 2010; Laakasuo et al., 2016). However, most previous research on friendship and personality considers relationship dyads, and the group dimension remains much less explored (Moreland and Levine, 2006; Forsyth, 2010) with an except for demographic effects (David-Barrett, 2019). Here, we are interested in personality homophily in close friendship groups.

### Personality Homophily in Groups

Homophily in personality has been suggested to serve two main functions. First, it may reduce cognitive load: if other individuals act and react similarly to oneself, less cognitive effort has to be spent in predicting their behavior (Leary, 1957; Brashears, 2013; Machin and Dunbar, 2016; van Duijn, 2016). Second, if homophily is a widespread social choice criterion, it can increase network density (David-Barrett and Dunbar, 2017). Personality homophily is also found in other social species (Seyfarth and Cheney, 2012): for instance, captive chimpanzees prefer others who are similar in boldness and sociability (Massen and Koski, 2014) and domestic horses prefer individuals of similar boldness and rank (Briard et al., 2015).

Earlier studies on friendship dyads found that extraversion, agreeableness and openness to experience can predict friendship formation in adolescents and young adults (Nelson et al., 2011), whereas no effect was found for neuroticism or conscientiousness (Selfhout et al., 2010; see also Kurtz and Sherker, 2003). Although similarities in personality can affect group behavior in different ways (van Knippenberg and Schippers, 2007), it is understudied for friendship groups (Liu et al., 2015). However, the Five Factor model of personality has been quite extensively used to study group efficiency in organizational settings (Barrick et al., 1992; Peeters et al., 2006; Prewett et al., 2009; LePine et al., 2011). Results show that personality differences in a group can affect behavioral coordination, social competition, and social structure at large (O’Connor and Dyce, 1997; Smith et al., 1999; Wolf and Krause, 2014). Especially conscientiousness has often been associated with group efficiency and performance (e.g., Barrick and Mount, 1991; Ilies et al., 2009; Chiaburu et al., 2011; Hill et al., 2014). Moreover, personality variance is of special interest for group functioning (Halfhill et al., 2005; Ferguson and Peterson, 2015; Liu et al., 2015). The review by van Knippenberg and Schippers (2007) found beneficial effects of both diversity and similarity of different personality traits: group diversity can be detrimental for prosocial personality traits such as conscientiousness and agreeableness (Peeters et al., 2006), but beneficial for proactive traits such as extraversion and openness (Chiaburu et al., 2011; French and Kottke, 2013; Kramer et al., 2014; Sung et al., 2014). Also variation in emotional stability (neuroticism) has been related to group sociality and performance (e.g., Trimmer et al., 2002).

### The Present Study

Here, we are interested in group-level variation in personality and functioning in friendship groups. We investigate two research questions:

(1) Do individuals in our sample tend to cluster in friendship groups around some personality traits, thus exhibiting homophily in group formation? We predict that personality homophily will affect group formation, so that *individuals will prefer to team up with individuals who are similar to them in personality* (H1).
(2) Is group success related to group-level variation in personality traits among friendship groups? More specifically, we ask whether (i) group performance/identification and (ii) group bonding are related to group-level variation in personality traits. We predict that group success will relate to personality traits, so that *group diversity in conscientiousness and agreeableness (prosocial traits) will be detrimental for group success* (H2) but that *group diversity in extraversion (proactive trait) may be beneficial for group success* (H3).

We also contribute to the field of personality and small group studies by suggesting a new data-transformation or analysis method in order to investigate homogeneity of the individual in comparison to their group members. This method differs from observing the personality variance on group level (used by, e.g., Peeters et al., 2006; Bell, 2007), and allows researchers to avoid complicated group clustering models and observe meaningful patterns in average-sized data sets in a simple manner.

### Fraternity Friendship Study

The data stems from a real-world case study of human friendship groups formed in the context of a university fraternity in Europe. Friendships established during the formative years of early adulthood are arguably among the most crucial during the whole lifespan (e.g., Kalmijn, 2003; Selfhout et al., 2010). As far as we are aware, this is the first exploration of how the variation in Big Five personality traits are involved in group formation and group success among close friends in a real-life setting (for work or study teams or in laboratory settings see Peeters et al., 2006; LePine et al., 2011).

The data used here were collected as part of the Fraternity Friendship Study, a longitudinal study of group formation conducted in an old and large (currently ∼1700 members, with 300–400 new members each year) student organization (‘Fraternity’) at a major European university^1^. Every year, the new members are encouraged to form smaller groups, henceforth called ‘friendship groups,’ each consisting of around 5–20 members. First, in August, prospective members engage in various bonding activities, ranging from sports tournaments and games to cleaning up the Fraternity building or working in the student restaurant. Starting in mid-September they start forming friendship groups with others in their intake year. Group formation is mostly ‘bottom–up, but also partly ‘top–down’ and overseen by a special committee of the fraternity. Groups are typically same-sex. After 8–9 weeks, groups can register themselves as a named entity and seek approval from the Fraternity board. Group membership is exclusive and, once formed, fixed. Friendship groups meet 1–3 times weekly. They showcase their identity through distinctive signals including their name, logo, certain colors and clothing (a tie, a skirt), their own songs and dances, or a website (Pearce et al., 2016^1^). Created in this way, Fraternity members come to feel very close to their group within the first year. Friendship groups usually go on for much longer than individuals participate in general fraternity activities and often create lifelong friendship bonds. Group members are often invited to each other’s weddings and doctoral defenses, and go for weekend getaways on a yearly basis, or longer trips for their 5^th^, 10^th^, or 15^th^ year anniversaries (Lehtonen et al., 2018).

### Data Collection

We conducted a baseline survey of new recruits at the start of the academic year, before formation of the friendship groups (September: wave 1). The baseline survey included questions about socio-economic background, lifestyle (hobbies, sport, health-related behavior), social life (friends and dating), life goals, affectual state and personality. We then inquired at the end of the first term (December: wave 2) and the end of the academic year (June: wave 3) about social relations and group performance. The questionnaires were in the participants’ native language and distributed online in cooperation with the Fraternity board. Participation was encouraged through small rewards, e.g., a cake. Study participants were informed about the purpose of the study and the conditions for participating. The survey and data storage plan have received ethical approval from the University of Helsinki (Statement 10/2014).

The full cohort included 387 new prospective members. These members formed 26 friendship groups (16 female groups), for which we use fictive names. Response rates were 52–57%: from the total of 387 students, 221 (140 females) responded in the first survey in the autumn, 210 (141 females) responded in the second survey, and 198 (136 females) responded in the third survey. Friendship groups on average participated in 1.7 out of 3 surveys (minimum: 0,9, maximum: 2,6, mean standard deviation: 1,17). Mean response rates for male groups were in 1.4 surveys (*SD:* 1.2) and for female groups at 1.8 (*SD:* 1.7). 146 students responded both in wave 1 and wave 3 and 136 students responded in all the three survey waves (Table 1).

**TABLE 1:** Descriptive statistics and factors associated with group formation (*N* = 221; Wave 1).

				**Proportion of variance**
	**All (*N* = 221)**	**Females (*N* = 140)**	**Males (*N* = 81)**	**explained by group**
**Variable (scale)**	**Mean (SD) or%**	**Mean (SD) or%**	**Mean (SD) or%**	**clustering (ICC)**
Age	18.0 (1.1)	17.9 (0.9)	18.2 (1.3)	0
Extraversion	3.7 (0.5)	3.75 (0.5)	3.6 (0.5)	0.06^+^
Agreeableness	3.6 (0.5)	3.6 (0.5)	3.6 (0.5)	0
Openness to experience	3.4 (0.5)	3.4 (0.5)	3.4 (0.5)	0.04
Conscientiousness	3.2 (0.6)	3.3 (0.6)	2.9 (0.5)	0.18***
Neuroticism	2.85 (0.6)	3.0 (0.5)	2.6 (0.6)	0.17***
Life satisfaction (1–10 ascending)	7.6 (0.9)	7.6 (1.0)	7.6 (1.0)	0.05^+^
Negative affect (4–20 ascending)	8.2 (2.3)	8.56 (2.4)	7.5 (2.0)	0.06^+^
Life goals: having an exciting lifestyle (yes)	22%	20%	25.5%	0.17***
Life goals: having a prestigious occupation (yes)	29%	32%	22.3%	0.08**
Health (1–5 descending)	2.7 (0.9)	2.6 (1.0)	2.9 (0.9)	0.08*
Smoking (1–7 ascending)	5.5 (1.9)	5.8 (1.7)	4.9 (2.2)	0.24***
Frequency of alcohol use (1–7 descending)	3.5 (0.8)	3.2 (0.7)	3.8 (0.8)	0.13**
Family member had a long-term illness (yes)	23%	23%	23.5%	0.09*

***p < 0.001, **p < 0.01, *p < 0.05, ^+^*p* < 0.10. ICC values are not mutually adjusted. The analysis has also been separately run for both sexes.

### Measures Used

The first survey asked questions related to social life and childhood experiences, self-rated general health (‘In general, would you say your health is,’ scale 1 ‘excellent’ to 5 ‘poor’), smoking frequency (‘How often do you smoke?’ scale 1 ‘never’ to 7 ‘every day’) and alcohol use (‘How often do you usually drink alcohol,’ scale 1 ‘every day’ to 7 ‘never’). *Personality* was measured through the short version of the Big Five assessing agreeableness, extroversion, openness, neuroticism, and conscientiousness (John and Srivastava, 1999); traits which are stable in young people and adolescents, too (Elkins et al., 2017). *Life Goals* were assessed with the Major Life Goal inventory (Roberts et al., 2004). *Individual well-being* was measured with different questions including life satisfaction and positive affect. *Life satisfaction* was measured with the question ‘All things considered, how satisfied are you with your life as a whole these days?’ (scale from 1 ‘extremely dissatisfied’ to 10 ‘extremely satisfied’). *Positive and Negative affect* was assessed with 6 of the original 8 items in the Affect Scale (Mroczek and Kolarz, 1998), and namely: during the past 30 days, how much of the time did you feel…(Scale: 1 All of the time to 5 none of the time) ‘cheerful?,’ ‘calm and peaceful?,’ ‘satisfied?,’ ‘full of life?,’ ‘so sad nothing could cheer you up?,’ ‘restless or fidgety?,’ ‘that everything was an effort?,’ ‘worthless?’ Questions directly measuring negative affect were items 5–8, which were combined into one measure of Negative Affect (see Table 1).

The final analyses focused on personality, group formation and group success. *Group formation* was measured as how individuals clustered in friendship groups. *Group success* was measured in three ways, *group performance/identification, group bonding*, and *group size.* Previous research has suggested that the level of commitment to one’s group affects the group’s performance (Mullen and Copper, 1994). We measured *group performance (or identification)* as the sum of all different group markers adopted by each individual within their friendship group. A continuous variable was created counting all ways the individual had adopted group markers in Wave 3 (conducted at the point in time when most such group markers – ties, socks, skirts, a song, a logo, etc. – were adopted). Mean number of adopted group markers in our data was for all individuals 4.89 (*SD:* 1.35 [range of SD for sub-groups ∼ 0.5–1.9], minimum: 1, maximum: 8), females was 4.94 (*SD*: 1.38, minimum: 0, maximum: 8) and males 4.79 (*SD:* 1.28, minimum: 3, maximum: 8). *Group bonding* was measured in wave 3 by how close members felt to the group (inclusion of other in self-visual scale, scale of 1 to 7; Aron et al., 1992), mean closeness for all respondents was 5.43 (standard deviation 1.27 [range of SD for sub-groups ∼ 0.5–2.0], minimum: 1, maximum: 7), females 5.58 (*SD:* 1.28, minimum: 1, maximum: 7) and males 5.04 (*SD:* 1.28, minimum: 3, maximum: 7). Correlation between group performance and group bonding was 0.21 (0.29 for males and 0.13 for females; sharing only about 4% of variance), indicating that these measures do tap into different dimensions of group success. We also ran an analysis by using personality dimensions as predictors (Wave 1) predicting the likelihood of answering the Wave 3 questionnaire. Only agreeableness had a slight effect on the likelihood of answering the follow-up questionnaire, which was to be expected (*B* = −0.73; *Z* = −2.08; *p* = 0.04).

### Statistical Analysis

**FIGURE 1:**
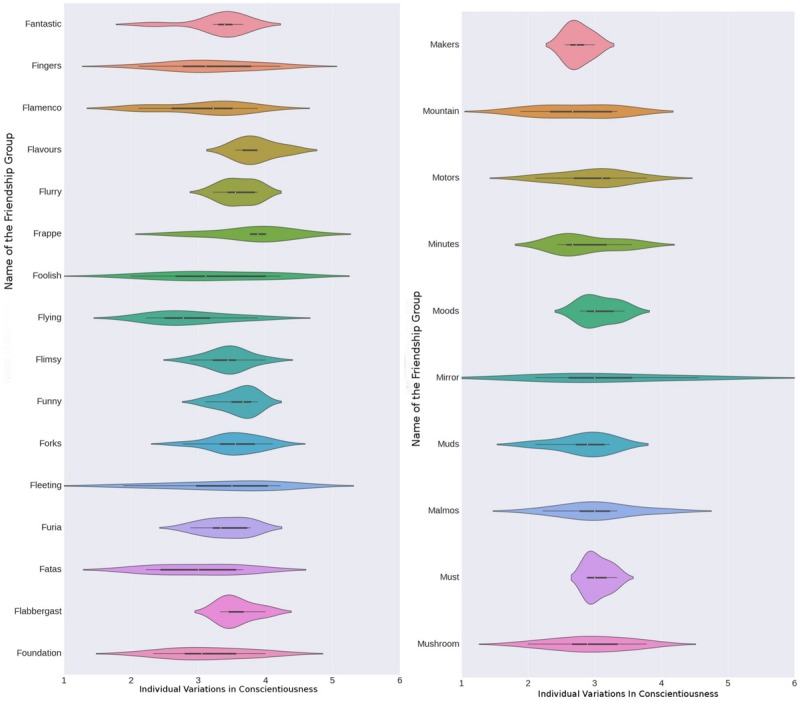
Variations in Conscientiousness for friendship groups, standard deviations for female groups (**left**, *N* = 16) and 10 male groups (**right**, *N* = 10).

To investigate associations between individual characteristics and *group formation*, multilevel regression analyses and one-way analyses of variance were conducted. We report intra-class correlations (ICC), showing how much of the variance in a trait is explained by variance between groups (Table 1 above). Figure 1 illustrates the data by showing variations for each friendship group with regard to conscientiousness. Group names are fictionalized.

**FIGURE 2:**
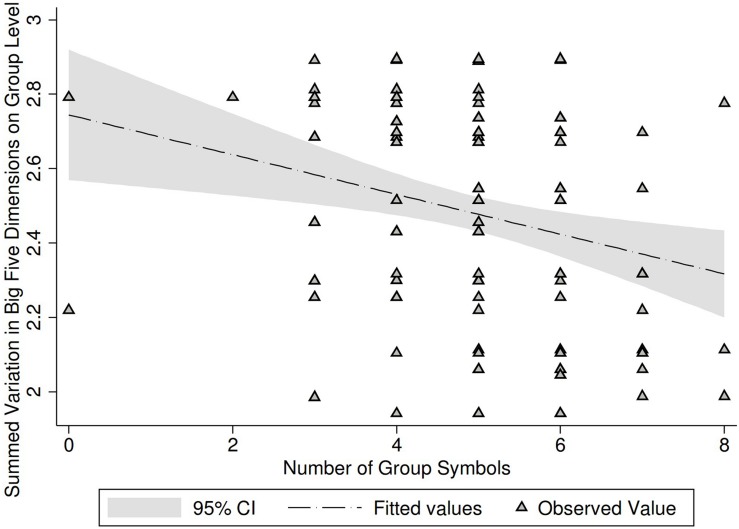
Association of group variation in Big Five and group performance. Personality measured as standard deviation at the level of ego’s group membership. Group performance/identification measured as number of group symbols acquired by each group member. Regression controlling for age and gender.

**FIGURE 3:**
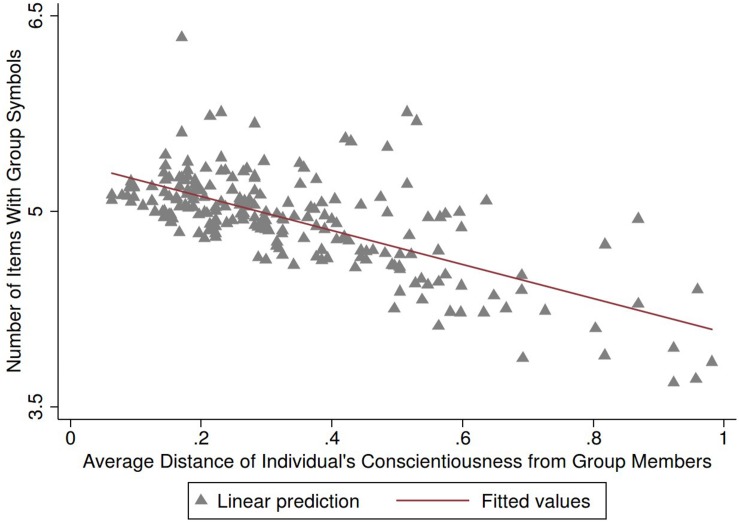
Association of variation in group conscientiousness and group performance/identification. Personality measured as individual’s distances to other group members taking into account heteroscedasticity. Group performance/identification was measured as number of group items adopted. Regression controlling for gender and individual’s own personality traits.

**FIGURE 4:**
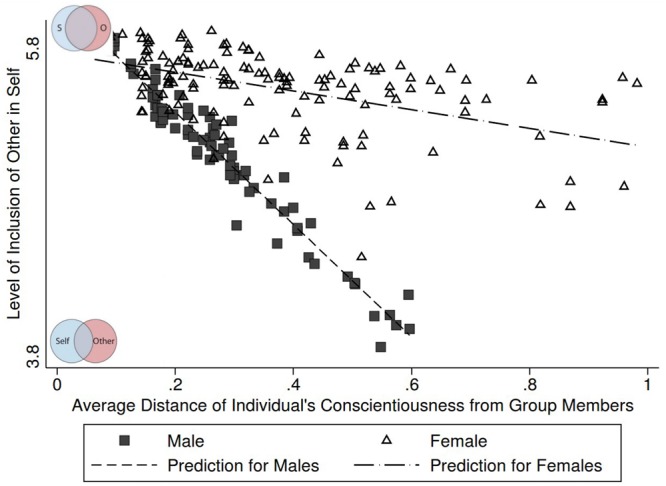
Associations of variation in group conscientiousness and group bonding in male and female friendship groups. Personality measured as individual distance to other group members for male and female groups taking into account heteroscedasticity. Group bonding measured through the Inclusion of Other in Self-scale.

To assess effects of group variation on *group success* (group performance and group bonding), we used two different and complimentary methods allowing us to investigate within-group variations of a certain personality trait (van Knippenberg and Schippers, 2007; Keiser et al., 2014, p. 847); we will also use the second method to see if it predicts group size (see below). First, the so-called variance method entailed calculating standard deviation for selected measures on a group level and assigned the resulting value to each individual in that group. All group members acquired one value, which did not differ within groups (see Prewett et al., 2009; see also Kramer et al., 2014 and Table 2). Values were regressed against our dependent variables that had individual-level variation. This method was also used for creating aggregate group-level variation for each personality trait combined into one BIG 5 variable (Figure 2). Second, for each participant, we created a new variable that indicated the individual’s average distance from other members in his or her group (ego’s aggregated distance from each member of the group divided by number of group members, not including ego^2^). Every respondent was assigned one individual value for each personality trait, five values in total. This second type of analysis was done with robust regression methods using heteroscedasticity adjustments for error terms (HC2 and HC3; where appropriate) (see Table 3 and Figures 3, 4 below); different adjustments did not alter the main results although they somewhat reduced statistical significance. This creates a measure of homogeneity for the *individual* with respect to his/her group members, and allows us to simplify the regression models by excluding group level clustering from our model. Both methods of analysis converge on similar interpretations (see sections “Results” and “Discussion”), but in our estimation, the second method is superior in clarity and simplicity. Regarding power analysis, the model does not need special modifications due to complexities stemming from number of groups, since we ‘flattened’ the data by concentrating on how much individuals differ from their group members. In our most complicated model, we have 12 predictors and if we assume a conservative effect-size (R^2^ of about 0.15 or *Cohen’s f^2^* of about 0.18), we need about 130 individuals for a power of about 0.9^3^; all our analyses include over 130 individuals. The sample size of 164 gives us power of 0.75 for smaller effect sizes in the range of 0.09–0.10. Analyses were conducted using STATA 13.1 software.

**TABLE 2:** Group performance, personality traits, and combined variation in Big 5, *N* = 149; univariate and multivariate regressions.

	**Simple model**			**Mutually adjusted model**		
	**(Univariate regressions)**			**(Multivariate regression)**		
**Variable**	**β**	**95% CI**	***p***	**β**	**95% CI**	***p***
Individual extraversion	0.09	−0.06, 0.25	0.25	0.11	−0.08, 0.24	0.33
Individual agreeableness	0.00	−0.15, 0.17	0.91	−0.03	−0.19, 0.12	0.65
Individual openness	−0.11	−0.27, 0.05	0.18	−0.10	−0.26, 0.06	0.24
Individual conscientiousness	**0.16**	0.01, 0.33	0.04*	0.08	−0.05, 0.28	0.19
Individual neuroticism	−0.13	−0.29, 0.02	0.11	−0.15	−0.32, 0.02	0.08^+^
Aggregate group level variation for all personality traits	−**0.24**	−0.40, −0.08	0.03*	−0.24	−0.40, −0.07	0.005**

**p < 0.01, *p < 0.05, ^+^*p* < 0.10. Variation in personality traits measured as standard deviation from group mean. Beta values are standardized regression coefficients. All regressions control for individual’s age and gender. Statistically significant values have been highlighted in bold.

**TABLE 3:** Group performance and personality variation, *N* = 164.

	**Simple model**			**Mutually adjusted model**		
	**β**	**CI**	***p***	**β**	**CI**	***p***
Variation in extraversion	−0.06	0.22, 0.08	0.38	−0.00	−0.20, 0.18	0.93
Variation in agreeableness	0.07	−0.07, 0.23	0.32	0.09	−0.11, 29	0.38
Variation in openness	−0.09	−0.24, 0.06	0.24	−0.12	−0.29, 0.04	0.15
Variation in conscientiousness	−**0.19**	−**0.35**, −**0.04**	0.01*	−**0.28**	−**0.54, 0.01**	0.04*
Variation in neuroticism	0.08	−0.07, 0.24	0.29	0.18	−0.05, 0.41	0.12

*p < 0.05. Variation in personality traits measured as average distance of individual to other group members taking into account heteroscedasticity. Group performance/identification measured as number of group symbols acquired by each group member in relation. Bivariate and mutually adjusted regression coefficients controlling for individual’s own personality, gender and age. Statistically significant values have been highlighted in bold.

## Results

### Factors Associated With Friendship Group Formation

The first research question was which variables, measured at the baseline survey at the start of the academic year, predicted which individuals chose to form friendship groups some months later. Table 1 shows the means of key individual characteristics and their association with group formation. Our first hypothesis was confirmed since similarity in personality was associated with group formation. Two personality traits, conscientiousness and neuroticism, were statistically significantly associated with group formation. No significant associations were found for agreeableness and openness, while a small association was detected for extraversion.

Among other variables measured, only a few sociodemographic and childhood family factors had any association with group formation. Individuals who had experienced long-term illness of a family member in their childhood were more likely to cluster in the same friendship group. Of life goals that respondents declared having for the future, having an exciting lifestyle and having a prestigious occupation related to group formation. Smoking and alcohol use also strongly predicted group formation, while weaker effects were found for general health, negative affect and overall life satisfaction. As could be expected, negative affect, low life satisfaction and neuroticism were all positively correlated (0.36–0.52). The other factors (e.g., alcohol use and wanting an exciting lifestyle) were not well-correlated with each other (correlations of 0.2 or smaller).

### Personality Variance and Group Success

The second research question was whether the composition of personality traits in a friendship group affected how the group functioned during its first year. Group success was assessed in three ways, as group performance/identification, as group bonding and as group size.

#### Group Performance/Identification

We first tested how variation in different personality traits were associated with group performance/identification, measured by number of personal items acquired with one’s group symbol on it (Table 2). Results from regression analyses are provided in Table 2, first as univariate regressions and then in a mutually adjusted regression model, which also controls for age and gender. Since interactions with personality traits and gender were not statistically significant, results for male and female friendship groups are shown together. In the simple regressions for standard personality traits, only individual’s conscientiousness was positively associated with higher group performance/identification. Students with high conscientiousness were more likely to use several group markers. In the mutually adjusted regressions (standard multivariate regressions), conscientiousness is no longer statistically significant, but neuroticism was negatively associated with group performance below the 0.1 *p*-value threshold.

Next, we tested the main second research question, or whether the combined measure of variation in personality traits was associated with group performance/identification (last row in Table 2 and Figure 2). We created this measure for each participant by aggregating their group level variations for all the personality traits. This combined variation method had a low alpha (0.50) as should be the case for Big-Five dimensions. The combined measure of variation in all Big 5 personality traits was significantly associated with group performance/identification in both univariate and multivariate regression models. This suggests that the greater the personality differences within friendship groups, the lower their group performance, measured as numbers of group identity markers developed, and also after taking into account the effect of individual personality traits (Figure 2).

Next, we examined associations of individual-level variation in separate personality traits and group performance/identification. Here, we used the measure of the individual’s averaged distance to other group members with regards to a specific personality trait, which measures individual’s homogeneity with respect to group members. Results indicate that the strength of these associations varied by personality trait (Table 3). Higher group performance/identification was significantly associated with higher group-level similarity in conscientiousness [β = −0.28, *p* < 0.05, *t*(145) = −2.00] (Table 3 and Figure 3), controlling for individual’s own personality and gender. Individual’s proximity to his/her group members in agreeableness, neuroticism and extraversion were not separately associated with the number of group symbols adopted while proximity in openness was close to reaching statistical significance.

We had hypothesized that group-level diversity in conscientiousness and agreeableness would be detrimental for group success but that diversity in extraversion could be associated with improved group success. Results indicated an effect only for conscientiousness, in the expected direction.

#### Group Bonding

As a second measure of group success, we investigated group bonding, measured as the association between individual reports of closeness to the group (the Inclusion-of-Other-in-Self or IOS scale) and differences in group-level personality. Using the first method of analysis or the aggregate standard deviations, we obtained the same general trend as for group performance/identification: the aggregate group level variation for all personality traits was positively associated with the sense of belonging (IOS β = −30, 95%CI [−0.63, 0.01] *p* = 0.06; results not shown in Table 4). Using the more robust method of analyzing individual proximity to group members, no significant results were detected for all groups together (results not shown). However, since there was significant interaction with gender for this measure (*p* = 0.01 for conscientiousness and *p* = 0.03 for neuroticism), we ran the regressions separately for male and female groups. Table 4 shows results from simple and mutually adjusted regressions. In the simple regressions, higher distance (heterogeneity) from group members in personality traits was generally negatively related to group bonding. The associations are statistically significant for conscientiousness in male friendship groups and neuroticism in female friendship groups. In the mutually adjusted model, only larger within-group variation in conscientiousness in male groups significantly predicted lower group bonding. Associations with the other four measured personality traits distances and bonding were not statistically significant in the adjusted model (Table 4).

**TABLE 4:** Group bonding and group heterogeneity in personality, *N* = 164.

				**Mutually adjusted**		
	**Simple model**			**model**		
**Variable**	**β**	***t***	***p***	**β**	***t***	***p***
**MEN**						
Variation in extraversion	–0.11	–0.72	0.47	0.06	0.36	0.71
Variation in agreeableness	–0.13	–0.79	0.43	–0.07	–0.38	0.70
Variation in openness	–0.09	–0.47	0.62	–0.05	–0.27	0.78
Variation in conscientiousness	−**0.59**	−**2.23**	0.03*	−**0.57**	**1.95**	0.05*
Variation in neuroticism	–0.14	–0.77	0.44	0.11	0.44	0.66
**WOMEN**						
Variation in extraversion	–0.11	–1.14	0.25	0.02	–0.16	0.84
Variation in agreeableness	–0.06	–0.69	0.49	0.04	0.53	0.69
Variation in openness	–0.05	–0.45	0.65	–0.02	0.13	0.89
Variation in conscientiousness	–0.11	–1.15	0.25	–0.11	1.34	0.18
Variation in neuroticism	−**0.19**	−**2.16**	0.03*	0.17	–1.40	0.29

*p < 0.05. Personality measured as individual distance to other group members taking into account heteroscedasticity. Group bonding measured with Inclusion of Other in Self-scale. Beta values are standardized regression coefficients. Model controls for age and individual levels of personality. Statistically significant values have been highlighted in bold.

Thus, a higher individual distance from one’s group members in conscientiousness appeared to be detrimental for male friendship group bonding. Given that we had enough power to predict the effects reported reliably, it is noteworthy that the general pattern observed and reported here is also corroborated by the bi-variate regressions. Figure 4 illustrates these associations separately for males and females.

#### Group Size

Finally we also ran the same homophily analysis by using the group-size as the dependent variable (*N* = 214). However, nothing was significant; we also did not find the group-size to be predictive of any of our other dependent variables.

## Discussion

How do personality traits shape collective behavior in close social groups? In the famous science fiction story *Nine Lives* (Le Guin, 1969/2016), the ideal team consists of nine clones of the same individual who vary only in their gender. This group proves superior to any group of ordinary humans both as a work team and a group of friends. However, although homophily is well-known to be the most prominent feature of human dyadic relations, little is known about its role in friendship groups. Here, we explored whether higher similarity in personality traits does actually pave the way for friendship group formation and success even after controlling for individual’s own personality.

Results indicate that group-level similarities in personality influenced both group formation and dynamics in friendship groups among young Western adults, in accordance with our first hypothesis. Individual neuroticism and conscientiousness predicted group formation, indicating that young adults tended to spontaneously team up with other individuals who were similar to them regarding these two personality traits. Two of three personality traits related to prosociality (conscientiousness, neuroticism, and agreeableness) were significantly associated with group formation in our data. With relation to group success, larger within-group variation in personality was associated with poorer outcomes, measured as the adoption of group identity markers and reported closeness to group members. When individuals differed more from their group members in personality, the group performed worse, independently of how separate individuals scored on this trait. Especially similar levels of conscientiousness predicted group success (group performance for all friendship groups and group bonding for male friendship groups). The more similar group members were in their conscientiousness, the better the group performed and the closer its members felt to each other, again controlling for individual personality. However, we found no significant associations for either agreeableness or extraversion regarding group success for either gender.

As far as we are aware, this is the first time group-level effects of the Big Five model of personality have been documented for human friends in a real-life setting (cf. Selfhout et al., 2010; Back and Vazire, 2015). Previous research has demonstrated that individual personality is important for group dynamics in many social species (Wolf and Krause, 2014). In our data, personality was a statistically significant predictor of group formation, unlike the many other social and demographic variables that were also included in our survey, confirming the importance of personality for groups.

These findings have implications for our understanding of both personality differences and the high preference for homophily between friends (cf. Laakasuo et al., 2016). At least in relatively small human groups, what matters is not so much individual conscientiousness, as the individual’s similarity in consciousness in comparison to the rest of the group. Our results are in line with previous research on professional team performance, which have suggested that conscientiousness homogeneity is a desired trait for working groups (e.g., Prewett et al., 2009); a group of hippies, or a group of soldiers, are happier together than they would be when mixed.

Our findings are also in line with some earlier studies on the effects of personality variation (see Beal et al., 2003; Hülsheger et al., 2009 for a meta-analysis), although the lack of standardized methodology or time intervals make comparisons difficult (e.g., Dorfman and Stephan, 1984; Greene, 1989). Selfhout et al. (2010) analyzed emergence of dyadic friendships in social networks and found that friendship was more prominent among individuals high on extraversion and agreeableness, however they did not study this in a group context. Neither of these two personality traits, well-known to increase sociality, were associated with group-level dynamics in our study, since only conscientiousness and in some aspects neuroticism related to group dynamics. It may be that, since extrovert individuals are more likely to have more friends and agreeable individuals to be chosen as friends (Selfhout et al., 2010), variation in these traits in dyadic relations is complementary within a group. It should also be noted that these traits were not observed to be detrimental on a group level.

The ultimate function of homophily among friends may vary depending on what level it is studied, e.g., the dyadic or the group friendship level. Human cooperation typically takes place in ‘bands’ of 10–20 individuals, who are all part of a larger community and also engage in dyadic and triadic interactions. We studied real-life, spontaneously formed groups with an average group size of 14. That is larger than the most effective group size of about five people (the size of most rock music bands and effective research groups; see Wilson, 1997), but close to one of the seminal numbers (15) in the layered structure of human social networks (Binford, 2001; Sutcliffe et al., 2012) and corresponding to the ‘sympathy group’ size (Buys and Larson, 1979). Psychological experiments (Launay and Dunbar, 2015) indicate that the span of homophily is related to network size: when groups appear to be too inclusive they grow large and homophily no longer occurs, suggesting that it is not only positive associations with a trait that cause homophily, but also a sense of the exclusiveness of a group. Thus group homophily may be especially important for the close friendship groups studied here, while other group dynamics may benefit bigger groups.

Why is similarity in conscientiousness so strongly related to group success? The ultimate reason may relate to the crucial role of timing and coordination in human sociality. Since human groups typically disperse in daytime and come together in the evenings, following a fission-fusion pattern, our ancestors faced the challenge of how to maintain contact and coordination with physically absent others (Pearce, 2014). From this perspective, the tendency to bond with others with similar conscientiousness levels may reflect the challenge of group coordination: when to meet, and how to fulfill certain collective planning and action tasks (Berg, 2016; Dunbar and Shultz, 2017). Which particular features conscientiousness promotes in groups remains a topic for future studies.

There were some striking gender differences in how conscientiousness affected friendship groups. Compared to females, male group bonding increased more sharply with increasing variation in levels of conscientiousness. This suggests a greater importance of personality homophily, or at least of homophily in conscientiousness, among males compared to females. Considering the male-hunting-group hypothesis (e.g., DeVore and Tooby, 1987), this seems to make sense, since similar orientation to group work ethic is tantamount to the success of the group. Primate research has investigated why males are more gregarious compared to females (e.g., Wrangham, 2000), and the bigger benefits of group formation males appear to acquire. However, in our study no gender differences were found in relation to within-group distances in personality and group performance. Notwithstanding, there is an alternative interpretation of our results. There is some evidence that women are more cooperative (Rand, 2017) and more altruistic than men (Rand et al., 2016; Brañas-Garza et al., 2018). This might have contributed to the result that similarity in conscientiousness predicts group bonding for men but not for women. Perhaps women tend to form groups more naturally than men (because they tend to be more altruistic and more cooperative) and they do not need conscientiousness^4^. However, more research on gender similarities and differences in the benefits of homophily on group behavior is warranted (David-Barrett et al., 2015), to properly address these issues.

Our study has several methodological advantages. First, it concerns a natural, real-life process of group formation among adults, which is rare. Second, personality was measured before group formation, indeed before most participants had met each other, ruling out the possibility of social influence of the group upon its members before baseline. Third, our analyses seem to reliably produce converging results with different methods. Furthermore, our new method for estimating individuals homogeneity compared to their group produced sensible results in a reliable manner with more clarity than the variance method often used (Prewett et al., 2009).

Among the limitations of this study is the relatively small sample size and that not all group members participated in all surveys. Moreover, 53 people know somebody from their group before they began their university education, which might have influenced the group formation to some extent. Replication with larger data, preferably from different locations, is needed. We used the number of group markers as a proxy index for group functionality, although the number of markers may be less important than the degree to which group members use these markers (e.g., frequency of wearing special outfits or updating website). Future research should look closer into alternative indices of group performance and identification.

We conclude that similarities in specific personality traits appear to boost group performance/identification and bonding in friendship groups^5^. Homophily through conscientiousness seems to be a key ingredient. In addition to expanding our understanding of the importance of homophily in human sociality, this highlights one possible reason for the existence of personality in humans. Personality similarity is likely to have been beneficial not just for relationship dyads, but also for mutual understanding and coordination in close peer groups.

## Data Availability Statement

The datasets generated for this study will not be made publicly available; a strict data gathering agreement was made with the participants in the study to protect their anonymity, which could relatively easily be compromised. Contrary to common psychological laboratory study data-sets, this field data-set is relatively small and does contain confidential information regarding individuals. For details and negotiations regarding data authentication and sharing, please contact AR, anna.rotkirch@vaestoliitto.fi.

## Ethics Statement

The studies involving human participants were reviewed and approved by the University of Helsinki, Ethical Review Board in the Humanities and Social and Behavioural Sciences. The participants provided their written informed consent to participate in this study.

## Author Contributions

ML and AR drafted the first manuscript. ML analyzed the data, wrote the algorithm for data transformations, and made the figures. MD collected the data. AR, MD, VB, MJ, and TD-B conceived the study idea. AR, MJ, MD, and VB designed the questionnaires. All authors helped to revise the manuscript for important intellectual content.

## Conflict of Interest

The authors declare that the research was conducted in the absence of any commercial or financial relationships that could be construed as a potential conflict of interest.

## Footnotes

1 www.theffs.nl

2 I.e., if the group had five members, ego’s distance was calculated to four other members. With a quick glance this method might seem equivalent to fixed effect group mean centering transformation, but it is not the same. We did something very different. Let’s assume that we have data like this, where the letter represents the participant and the number represents the participants value on some personality trait: [A:1, B:2,C:3, D:4,E:6]. The group mean would be 3.2; and mean centering values would be: [A:2.2, B:1.2, C:0.2,D:0.8, E:2.8]; where as the average distances would be [A:2.25, B: 2.0, C: 1.75, D: 2, E:3.5]. So, as is clear, this is something different from doing “fixed effects” transformation on our data and it is not equivalent to adding a categorical variable per group, since there is no fixed term that is added or subtracted to each participant. The number is an index number assigned to each individual regarding how similar they are on average to others in their in-group.

3 www.danielsoper.com/statcalc/calculator.aspx?id=1

4 We would like to thank our reviewer for this important observation.

5 Participants gave their personality profiles before they were divided to groups, indicating that group-membership did not affect their personality. Controlling for this variable did not alter our results.
